# Post Graduate Instruction

**Published:** 1889-11

**Authors:** 


POST GRADUATE INSTRUCTION.—No better evidences of the upward tendency in the medical profession exist than the increasing number of Post Graduate schools, and the success which attends them. It is now conceded that while Germany and France are well up in scientific and theoretical progress, that the United States surpasses all other countries in therapeutics and practice. No other country can furnish so many bold, self reliant practitioners as are found in the United States of America.

